# Cancer-Type Regulation of *MIG-6* Expression by Inhibitors of Methylation and Histone Deacetylation

**DOI:** 10.1371/journal.pone.0038955

**Published:** 2012-06-12

**Authors:** Yu-Wen Zhang, Ben Staal, Karl J. Dykema, Kyle A. Furge, George F. Vande Woude

**Affiliations:** 1 Laboratory of Molecular Oncology, Van Andel Research Institute, Grand Rapids, Michigan, United States of America; 2 Laboratory of Computational Biology, Van Andel Research Institute, Grand Rapids, Michigan, United States of America; University of Navarra, Spain

## Abstract

Epigenetic silencing is one of the mechanisms leading to inactivation of a tumor suppressor gene, either by DNA methylation or histone modification in a promoter regulatory region. Mitogen inducible gene 6 (*MIG-6*), mainly known as a negative feedback inhibitor of the epidermal growth factor receptor (EGFR) family, is a tumor suppressor gene that is associated with many human cancers. To determine if *MIG-6* is inactivated by epigenetic alteration, we identified a group of human lung cancer and melanoma cell lines in which its expression is either low or undetectable and studied the effects of methylation and of histone deacetylation on its expression. The DNA methyltransferase (DNMT) inhibitor 5-aza-2′-deoxycytidine (5-aza-dC) induced *MIG-6* expression in melanoma cell lines but little in lung cancer lines. By contrast, the histone deacetylase (HDAC) inhibitor trichostatin A (TSA) induced *MIG-6* expression in lung cancer lines but had little effect in melanoma lines. However, the *MIG-6* promoter itself did not appear to be directly affected by either methylation or histone deacetylation, indicating an indirect regulatory mechanism. Luciferase reporter assays revealed that a short segment of exon 1 in the *MIG-6* gene is responsible for TSA response in the lung cancer cells; thus, the *MIG-6* gene can be epigenetically silenced through an indirect mechanism without having a physical alteration in its promoter. Furthermore, our data also suggest that *MIG-6* gene expression is differentially regulated in lung cancer and melanoma.

## Introduction

Mitogen inducible gene 6 (*MIG-6*) (also known as gene 33, *ERRFI1*, or *RALT*) is an immediate early response gene that is expressed in various tissues and plays a critical role in many patho-physiological states [1]. Its expression can be induced by a broad spectrum of growth factors, hormones, or stress stimuli, and it is associated with various chronic conditions [1], [2]. Studies in mice have revealed that *Mig-6* is required for skin morphogenesis and lung development and that it plays an important role in maintaining joint homeostasis [3], [4], [5].

As a cytoplasmic scaffolding adaptor, MIG-6 has several important protein-protein interaction motifs that may mediate interaction with signaling molecules downstream of receptor tyrosine kinases (RTKs) [2]. One of the most prominent roles of MIG-6 in regulating signal transduction comes from its ability to directly interact with epidermal growth factor receptor (EGFR) and other ErbB family members, inhibiting their phosphorylation and downstream signaling in a negative feedback fashion [6], [7], [8], [9]. MIG-6 can be induced by hepatocyte growth factor (HGF) and functions as a negative feedback regulator of HGF-MET signaling [10], [11], indicating that it has broad role as a signal checkpoint for modulating activated RTK pathways in a timely manner.

The evidence that *MIG-6* is a tumor suppressor gene is compelling. It is located in chromosome 1p36, a locus that frequently has loss of heterozygosity in several human cancers including lung cancer [12], [13], [14], melanoma [15], and breast cancer [16]. Indeed, down-regulation or loss of *MIG-6* expression has been reported in cancers and is often associated with poor prognosis [3], [11], [16], [17], [18], [19], [20], [21], [22], [23]. *MIG-6* down-regulation in non-small cell lung cancer (NSCLC) is associated with increased EGFR signaling and poorly differentiated cancer [21], while loss of its expression in ErbB2-amplified breast carcinoma renders the cancer cells more resistant to Herceptin, the neutralizing antibody against ErbB2 [16]. In glioblastoma, *MIG-6* is identified as a single gene within the most commonly deleted region at the 1p36.23 locus, and its expression is down-regulated in 34% of glioblastoma samples [19]. While *MIG-6* down-regulation is reported in a high percentage of papillary thyroid cancers [22], high *MIG-6* expression correlates with longer survival and is associated with favorable surgical outcomes for those patients [24]. Decreased MIG-6 expression has also been reported in skin cancer, endometrial cancer, and hepatocellular carcinomas [3], [20], [23]. Moreover, even though such events are rare, three mutations in the *MIG-6* gene have been identified in human lung cancer and one in neuroblastoma [11], [18]. Further evidence supporting *MIG-6* as a tumor suppressor gene arose from mouse studies; *Mig-6*-deficient mice are prone to develop epithelial hyperplasia or tumors in organs including the lung, skin, uterus, gallbladder, and bile duct [3], [11], [20].

Epigenetic alteration, one of the most well-known mechanisms leading to inactivation of a tumor suppressor gene [25], can result from DNA methylation or histone deacetylation in the gene's promoter [25]. Given that down-regulation of *MIG-6* is frequently observed in many human cancers, we asked whether *MIG-6* expression was affected by DNA methylation and histone deacetylation. Here, we show that the *MIG-6* promoter itself is neither hypermethylated nor affected by histone deacetylation. However, its expression is induced by the DNA methyltransferase (DNMT) inhibitor 5-aza-2′-deoxycytidine (5-aza-dC) in melanoma cell lines and by the histone deacetylase (HDAC) inhibitor trichostatin A (TSA) in lung cancer lines. By dissecting its promoter regulatory region using a luciferase reporter assay, we identified a minimal TSA-response element in exon 1 of *MIG-6* that is essential for its induction by TSA in lung cancer cells.

## Results

### MIG-6 expression is differentially regulated by 5-aza-dC in melanoma cell lines and TSA in lung cancer cell lines

**Figure 1:**
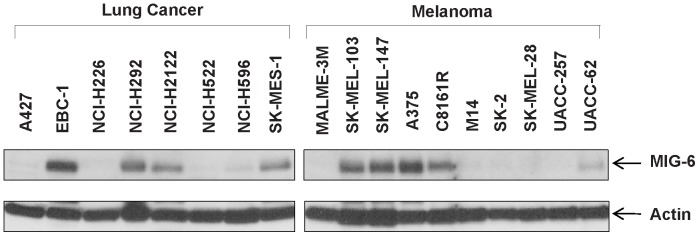
MIG-6 protein levels in lung cancer and melanoma cell lines. Whole cell lysates were prepared from the indicated cell lines, and MIG-6 was determined by western blot analysis using anti-Mig-6 polyclonal antibody. As a loading control, the same blot was probed with anti- β-actin antibody.

To determine whether *MIG-6* expression was affected by epigenetic alteration, we first identified human cancer cell lines in which its promoter is likely affected by methylation or histone deacetylation. As shown in Figure 1, we found four human NSCLC cell lines (A427, H226, H522, and H596) and five melanoma cell lines (M14, MALME-3M, SK-2, SK-MEL-28, and UACC-257) in which MIG-6 protein was either low or undetectable. We then treated these cell lines with or without 5-aza-dC, TSA, or a combination of both inhibitors.

**Figure 2:**
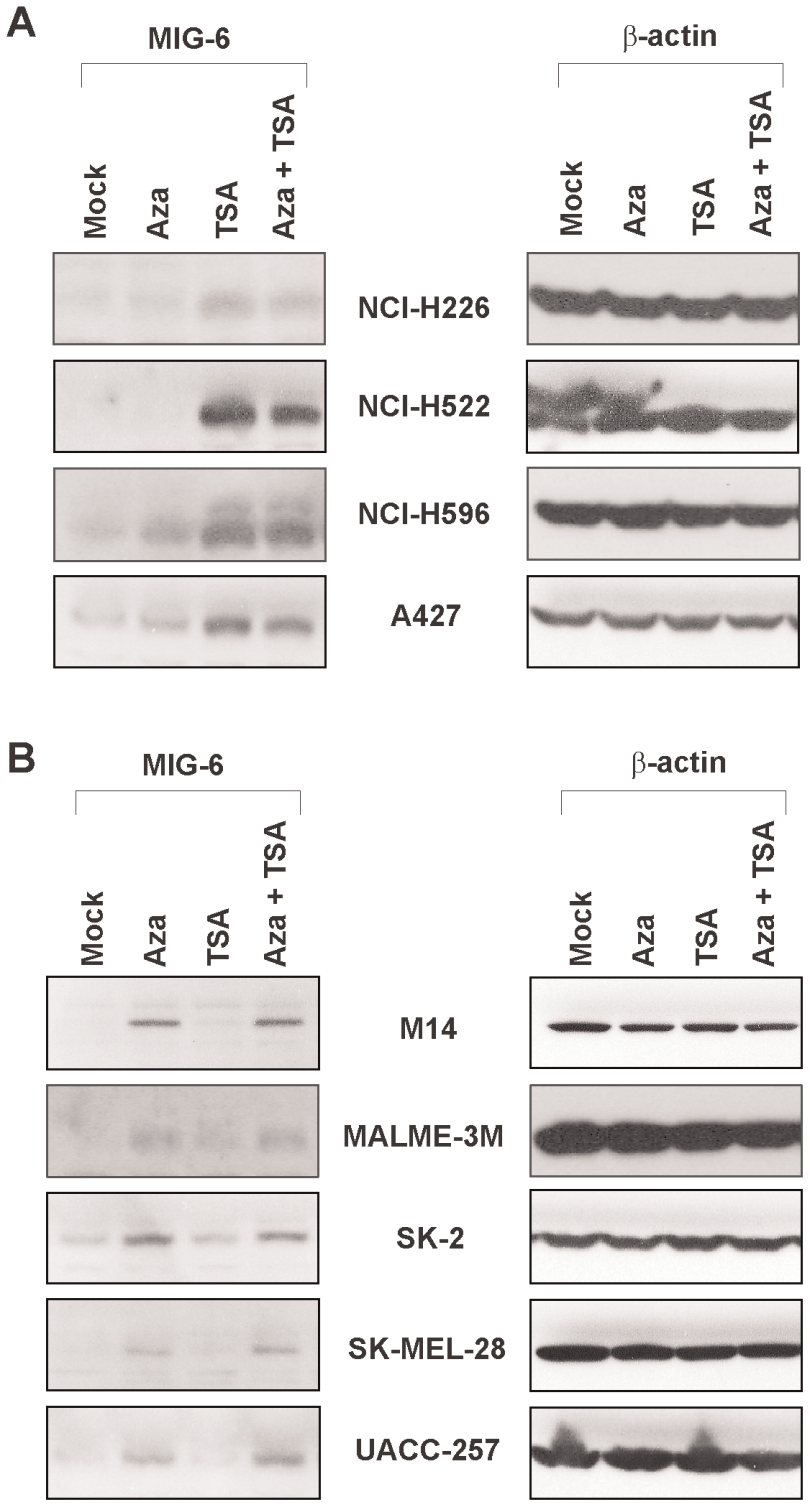
MIG-6 protein is differentially induced by 5-aza-dC and TSA in lung cancer and melanoma cell lines. Whole cell lysates were extracted from the cells treated with or without 5-aza-dC (10 µM) and/or TSA (1 µM), and western blot analyses were performed to detect MIG-6 protein. β-actin was used as an internal control. (A) MIG-6 protein was induced by TSA but not by 5-aza-dC in the lung cancer lines. (B) 5-Aza-dC, but not TSA, induced MIG-6 in the melanoma cell lines.

**Figure 3:**
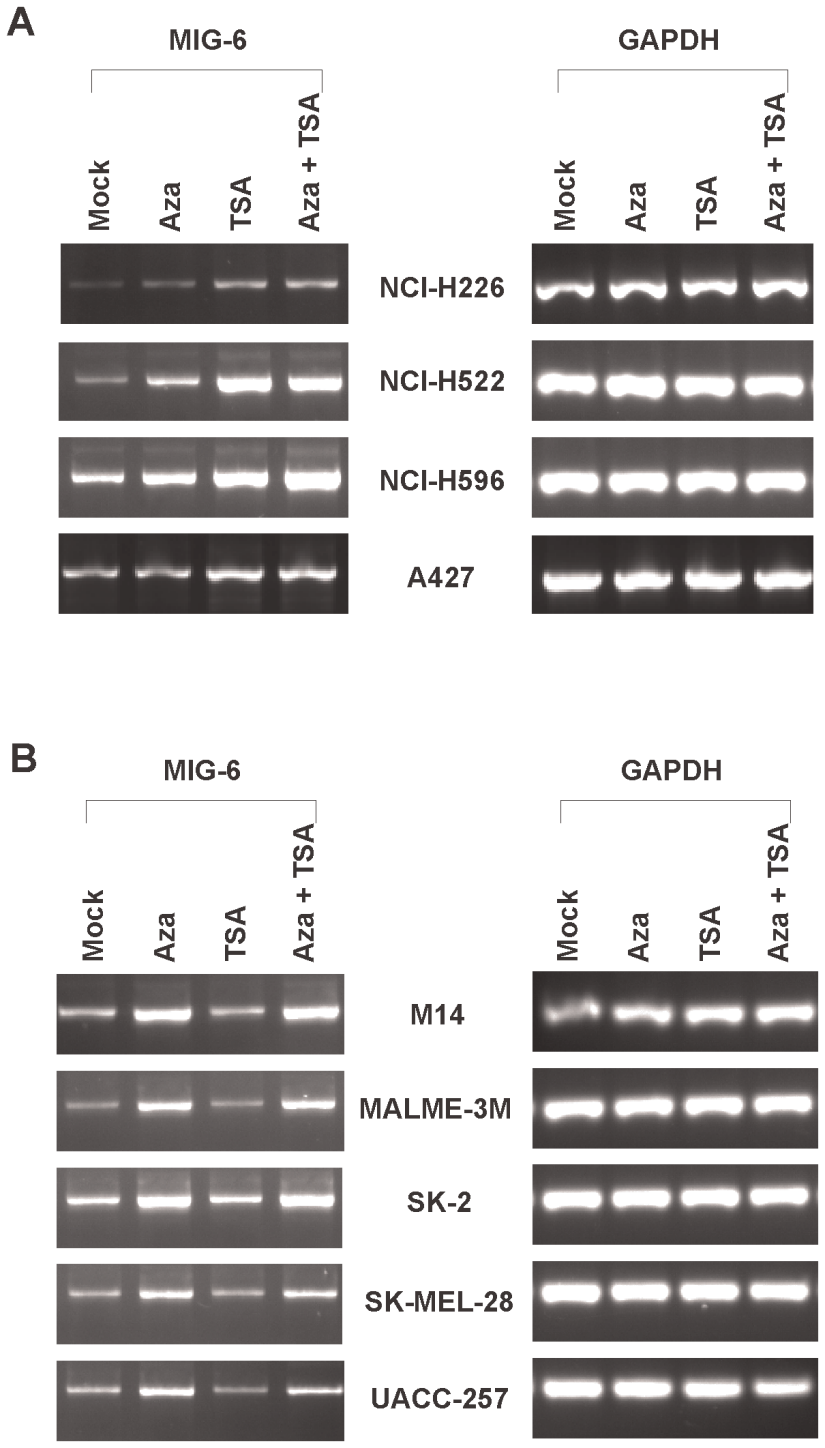
Induction of *MIG-6* expression by 5-aza-dC and TSA is regulated at transcriptional level. Total RNAs were isolated from cells treated with or without 5-aza-dC (10 µM) and/or TSA (1 µM), and *MIG-6* expression was determined by RT-PCR analyses. *GAPDH* expression was used as an internal control. (A) TSA increased *MIG-6* mRNA in the four lung cancer cell lines. (B) *MIG-6* mRNA in the five melanoma cell lines was increased by 5-aza-dC.

To our surprise, we found that TSA treatment significantly increased the amount of MIG-6 protein in the lung cancer cell lines, but not in the melanoma lines (Figure 2A). In contrast, 5-aza-dC treatment significantly increased the MIG-6 protein in the melanoma cell lines, but not in the NSCLC lung cancer lines (Figure 2B). To determine if the increase of MIG-6 protein was regulated at transcriptional level, we performed RT-PCR analysis. As shown in Figure 3, and consistent with protein expression, *MIG-6* mRNA expression increased with TSA treatment only in the four lung cancer cell lines, and it increased with 5-aza-dC treatment only in the five melanoma lines. These data strongly suggest that the induction of *MIG-6* expression by 5-aza-dC or TSA is regulated at the transcriptional level and is differentially regulated in the lung cancer and melanoma cells.

### The MIG-6 promoter is neither hypermethylated nor directly affected by histone deacetylation

**Figure 4:**
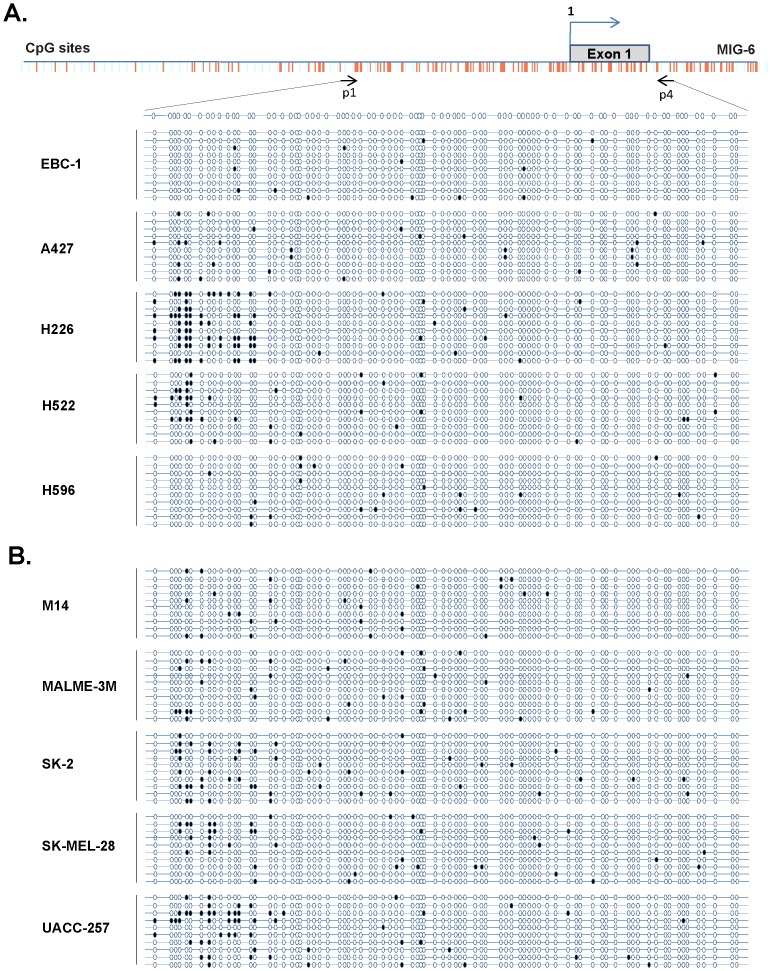
The *MIG-6* promoter is hypomethylated in lung cancer and melanoma cell lines. The *MIG-6* promoter was amplified from bisulfite-converted DNA and cloned into a TOPO TA-cloning vector. The status of *MIG-6* promoter methylation in (A) lung cancer cell lines and (B) melanoma cell lines was evaluated by sequencing 10 randomly picked colonies from each line for methylated cytosine residues. Each red bar represents a CpG site. The open ovals indicate unmethylated CpG sites and the solid ovals indicate methylated sites. EBC-1 cell line was used as a negative control to show the basal methylation status of MIG-6 promoter.

Given that *MIG-6* expression was induced by 5-aza-dC in the melanoma lines, we asked if its promoter was hypermethylated in those cells. We extracted genomic DNA from both lung cancer and melanoma cell lines and examined DNA methylation in the 596-bp *MIG-6* promoter regulatory region, which contains abundant CpG sites (Figure 4). To our surprise, the lung cancer cell lines (Figure 4A) and the melanoma cell lines (Figure 4B) were similar in having very few methylated CpG sites in the *MIG-6* promoter regulatory region, indicating that induction of *MIG-6* by 5-aza-dC in melanoma was independent of DNA methylation in its promoter. These results were confirmed by direct sequencing of the PCR products amplified from bisulfite-treated DNAs (data not shown).

**Figure 5:**
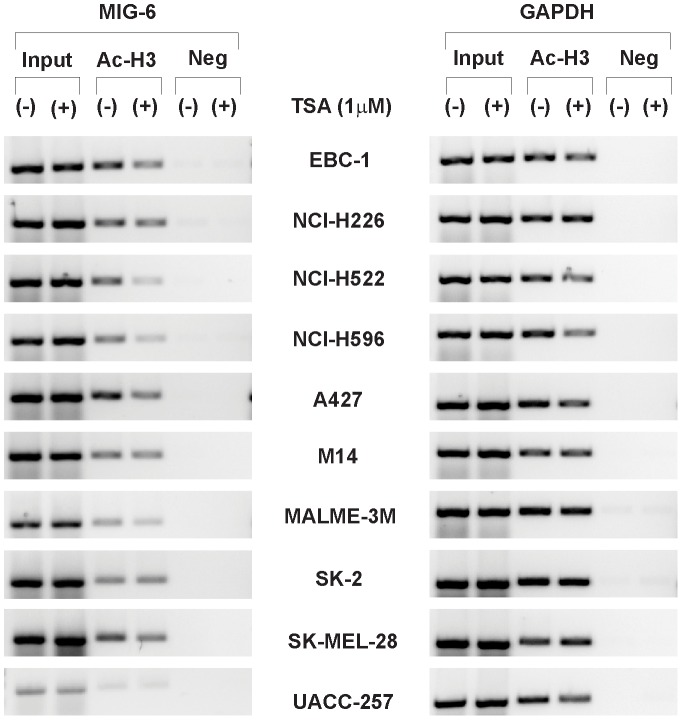
The *MIG-6* promoter is not affected by histone deacetylation. The cells were treated with or without TSA (1 µM) for 24 h, and a ChIP assay was performed using anti-acetyl histone H3 antibody. As a negative control, normal rabbit serum was used for immunoprecipitation. The DNA fragments cross-linked and co-immunoprecipitated with the acetylated histone H3 were purified and used for PCR amplification of the promoters of *MIG-6* and *GAPDH*. EBC-1 cell line was used as a negative control to show the basal histone deacetylation status of MIG-6 promoter.

Similarly, we asked if the *MIG-6* promoter was influenced by histone deacetylation. By chromatin immunoprecipitation assay (ChIP), we found that TSA treatment did not increase the binding of acetyl-histone H3 to the *MIG-6* promoter in the lung cancer lines or in the melanoma lines (Figure 5), indicating that the *MIG-6* promoter was not directly affected by histone deacetylation either.

### MIG-6 transcription is indirectly regulated by a factor(s) that is affected by methylation or histone deacetylation

**Figure 6:**
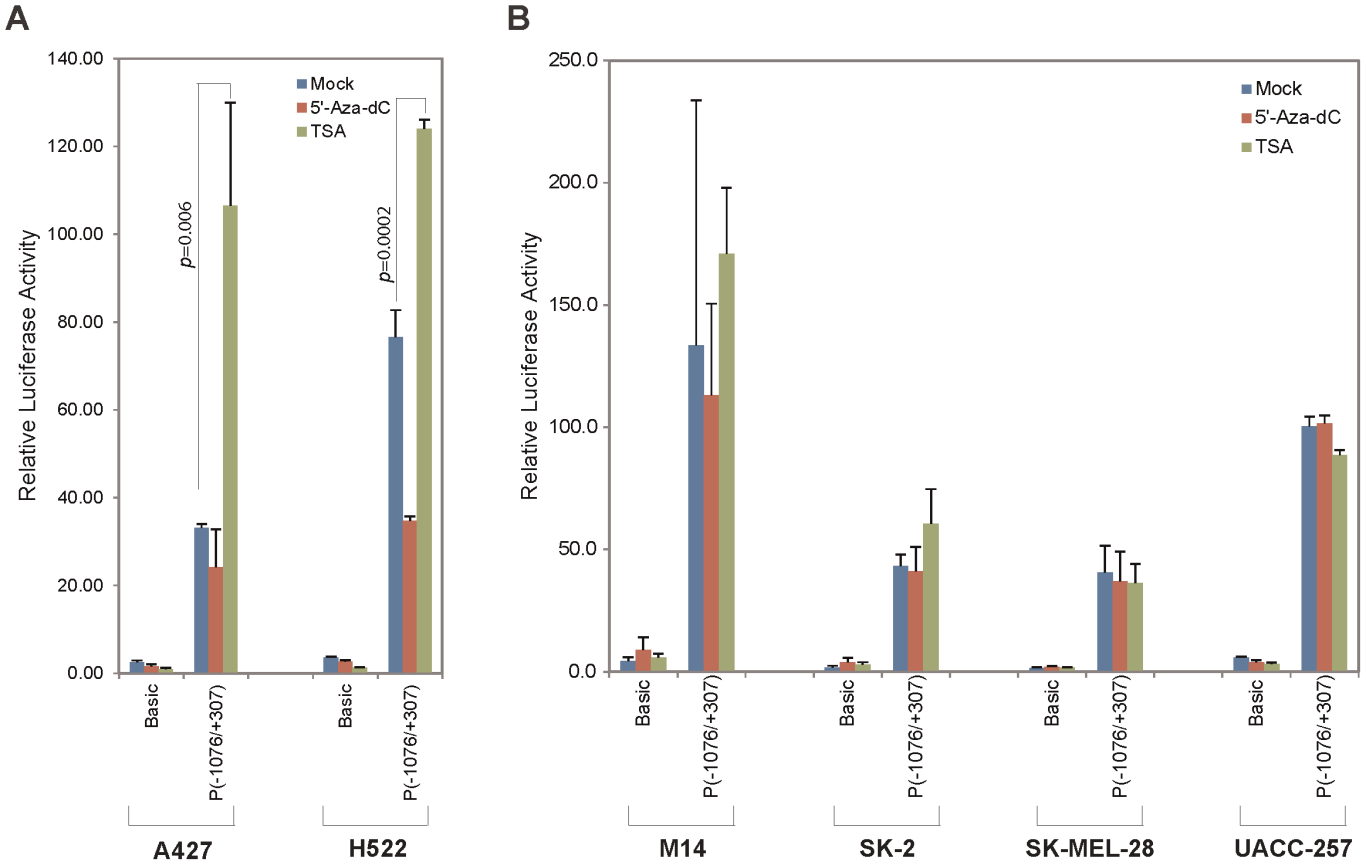
Determining 5-aza-dC- and TSA-response elements in the *MIG-6* promoter regulatory region. Lung cancer and melanoma cell lines were transiently transfected with luciferase reporter plasmids, either pGL3-Basic or pGL3-P(−1076/+307), followed by treatment with or without 5-aza-dC or TSA. The pU6B-*Renilla* reporter was co-transfected for normalization. Each assay was performed in triplicate. The error bars represent standard deviation. The student t-test *p* value indicates a statistically significant difference between the mock-treated and the TSA-treated samples.

Because the above data suggest that *MIG-6* induction is not directly regulated, we looked for a secondary mechanism, with the inhibitors inducing expression of a transcription factor(s) or co-factor(s) that in turn regulates *MIG-6* expression. Thus, we examined the responses of the *MIG-6* promoter regulatory region to the inhibitors via luciferase reporter assay. A *MIG-6* promoter reporter plasmid was constructed by inserting a 1.383-kb genomic DNA fragment (consisting of the *MIG-6* promoter, its upstream regulatory region, and the downstream exon 1 and part of intron 1) in front of a luciferase reporter gene. Testing the reporter in both lung cancer and melanoma cell lines, we found that TSA significantly enhanced *MIG-6* promoter activity in lung cancer cells but showed no such effect in melanoma cells (Figure 6). This data was consistent with our prior western blot and RT-PCR analyses. 5-aza-dC, however, appeared to have no effect on reporter activity in either the melanoma or lung cancer lines (Figure 6). These data indicate that while the TSA-responsive element is within the 1.383-kb region of *MIG-6*, the 5-aza-dC-responsive element is likely outside this region.

### A small segment of exon 1 in MIG-6 is essential for TSA response in lung cancer

**Figure 7:**
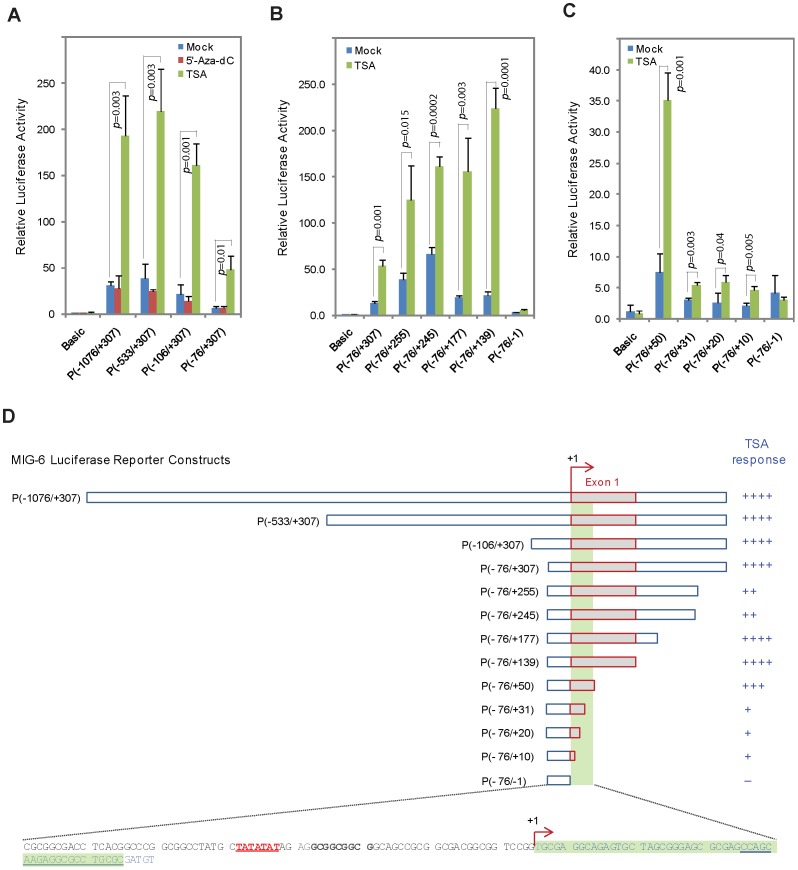
Mapping the TSA-response element in the *MIG-6* promoter regulatory region by deletion analyses. (A–C) Different lengths of the *MIG-6* promoter regulatory region were inserted into the pGL3 vector. The luciferase reporter assay was performed in A427 lung cancer cells transiently transfected with pGL3-Basic or the indicated reporter carrying *MIG-6* promoter element. The cells were then treated with or without 5-aza-dC or TSA. The pU6B-*Renilla* reporter was co-transfected for normalization. Each assay was performed in triplicate. The error bars represent standard deviation. (D) Schematic representation of the TSA-response element in the *MIG-6* promoter regulatory region. The arrow indicates the transcription starting site; the red box indicates exon 1. Shaded in green is the 50-bp element in exon 1 that is most likely responsible for TSA response in lung cancer cells, which we designated as the minimal TSA-response element.

We performed a series of deletion analyses in the 1.383-kb *MIG-6* promoter regulatory region to determine the minimal region required for induction by TSA in lung cancer cells. Deletion from the 5′-terminus to the proximal region of the *MIG-6* promoter resulted in a decrease of the basal promoter activity, while the response to TSA was essentially retained (Figure 7A). Deletion from the 3′-terminus to the transcriptional initiation site of *MIG-6* resulted in complete loss of response to TSA (Figure 7B), indicating that the TSA response element was downstream of the *MIG-6* promoter. Further deletion analyses revealed a small segment in the exon 1 starting from the transcriptional initiation site that was essential for TSA responsiveness (Figure 7C). As summarized in Figure 7D, the minimal TSA response element is within the first 50 nucleotides of exon 1, with the distal 20-nucleotide segment showing the highest activity.

**Figure 8:**
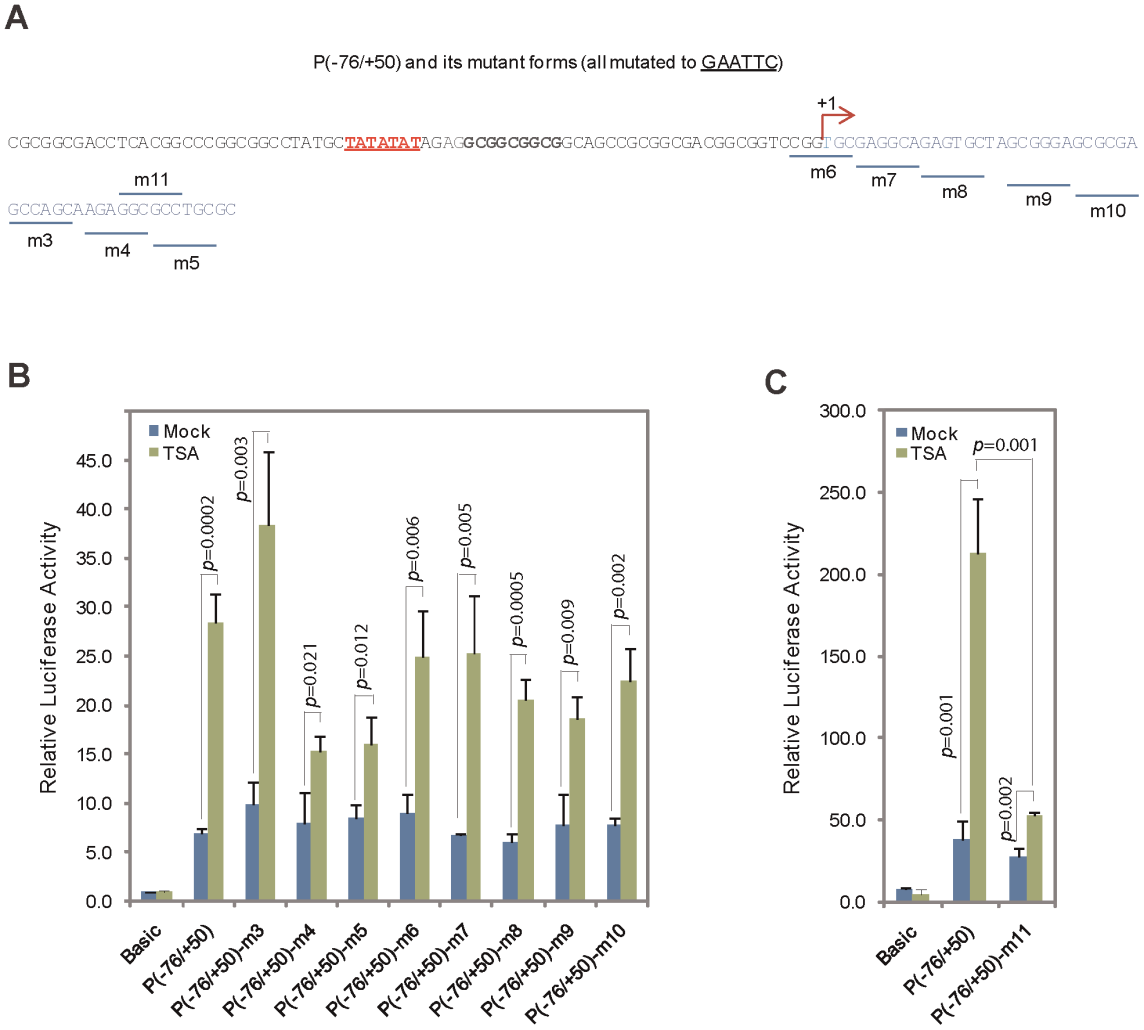
Dissecting the minimal TSA-response element in *MIG-6* by mutational analysis. (A) The sequences of P(−76/+50) that contain the *MIG-6* promoter and the minimal TSA-response element are shown. For each mutant reporter construct (m3-m11), the underlined sequence was mutated to GAATTC. (B and C) A luciferase reporter assay was performed in A427 lung cancer cells by transiently transfecting the indicated reporter plasmid with or without TSA treatment. The error bars represent standard deviation and all assays were performed in triplicate.

We speculated that there exists a critical transcription factor binding motif in the minimal TSA response element. We performed mutation analyses of the 50-nucleotide segment to pinpoint potential transcription factor binding motif(s) (Figure 8A). Compared with the wild-type P(-76/+50) reporter, mutation in the m4 and m5 elements resulted in a significant decrease of reporter activity in response to TSA, while mutation in other elements had lesser effect (Figure 8B). This result agrees with the deletion analyses, as the m4 and m5 elements are within the distal 20-nucleotide segment that, when deleted, resulted in a steep drop-off in TSA response. We generated another mutant reporter m11 in which half of the sequences in both m4 and m5 were mutated (Figure 8A). We found that the m11 mutant had a much greater reduction in TSA response (Figure 8C), indicating that those sequences are essential for the binding of a yet to be identified transcription factor which regulates MIG-6 gene expression induced by TSA in the lung cancer.

### Other genes differentially regulated by 5-aza-dC and TSA in lung cancer and melanoma cells

**Figure 9:**
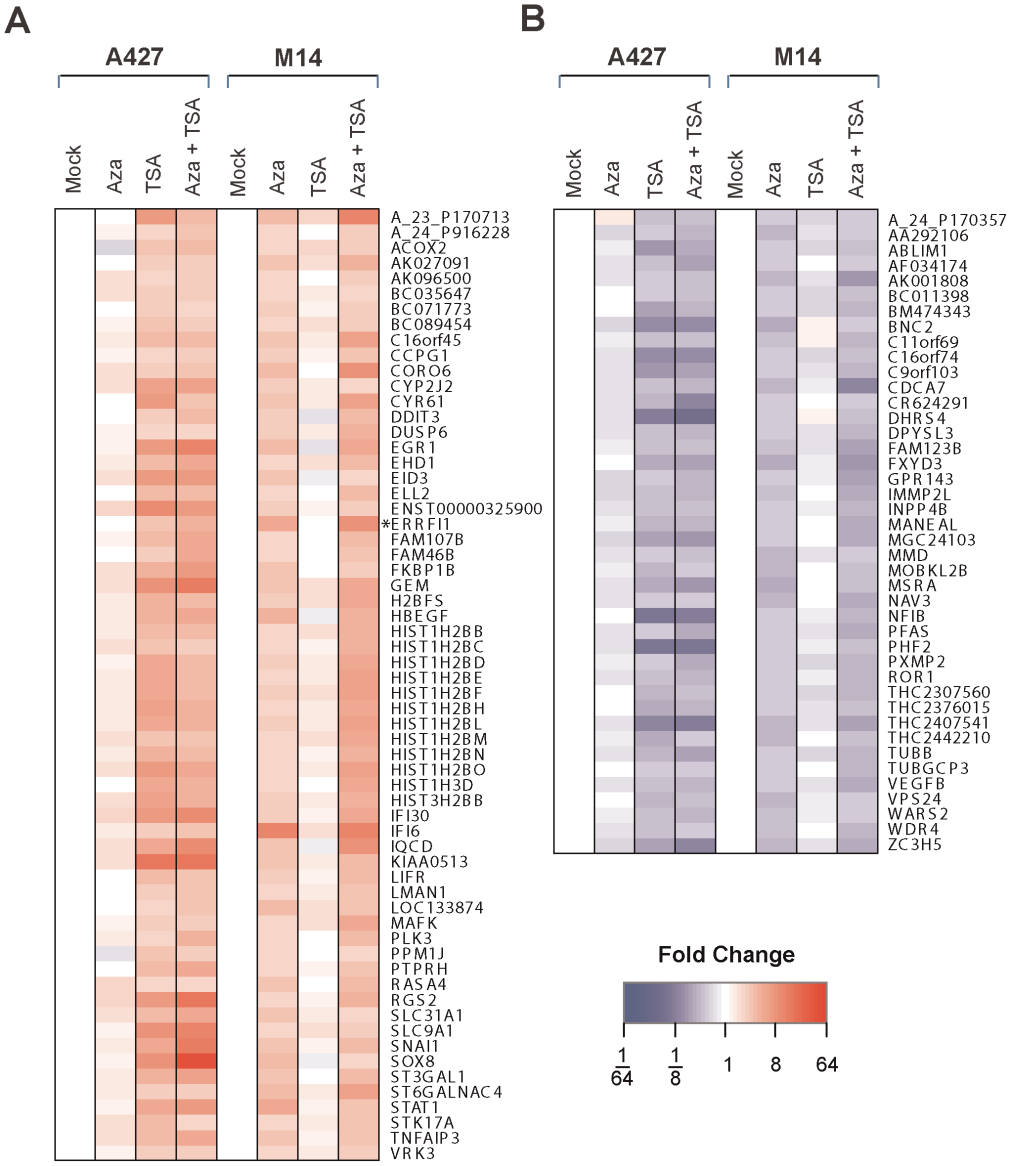
MIG-6 is not the only gene differentially regulated by 5-aza-dC and TSA in lung cancer and melanoma cells. Microarray analyses were performed on RNA samples from A427 lung cancer cells and M14 melanoma cells treated with or without 5-aza-dC (10 µM) and/or TSA (1 µM). The heat maps show (A) the genes whose expression pattern was similar to that of *MIG-6*, and (B) the genes whose expression was down-regulated by the treatment, in contrast to *MIG-6* expression. The *MIG-6* gene is indicated with an asterisk and is shown as the alternative symbol, *ERRFI1*.

We next asked whether there were other genes differentially regulated by 5-aza-dC and TSA in lung cancer and melanoma cells. We performed DNA microarray analyses on samples derived from A427 lung cancer and M14 melanoma cells treated with 5-aza-dC and/or TSA. Figure 9A shows the genes displaying an expression pattern similar to that of *MIG-6* (which is indicated as *ERRFI1* in the heat-map) in response to either 5-aza-dC or TSA treatment (Figure 9A). Another group of genes appeared to be down-regulated, the opposite of *MIG-6* expression (Figure 9B).

**Figure 10:**
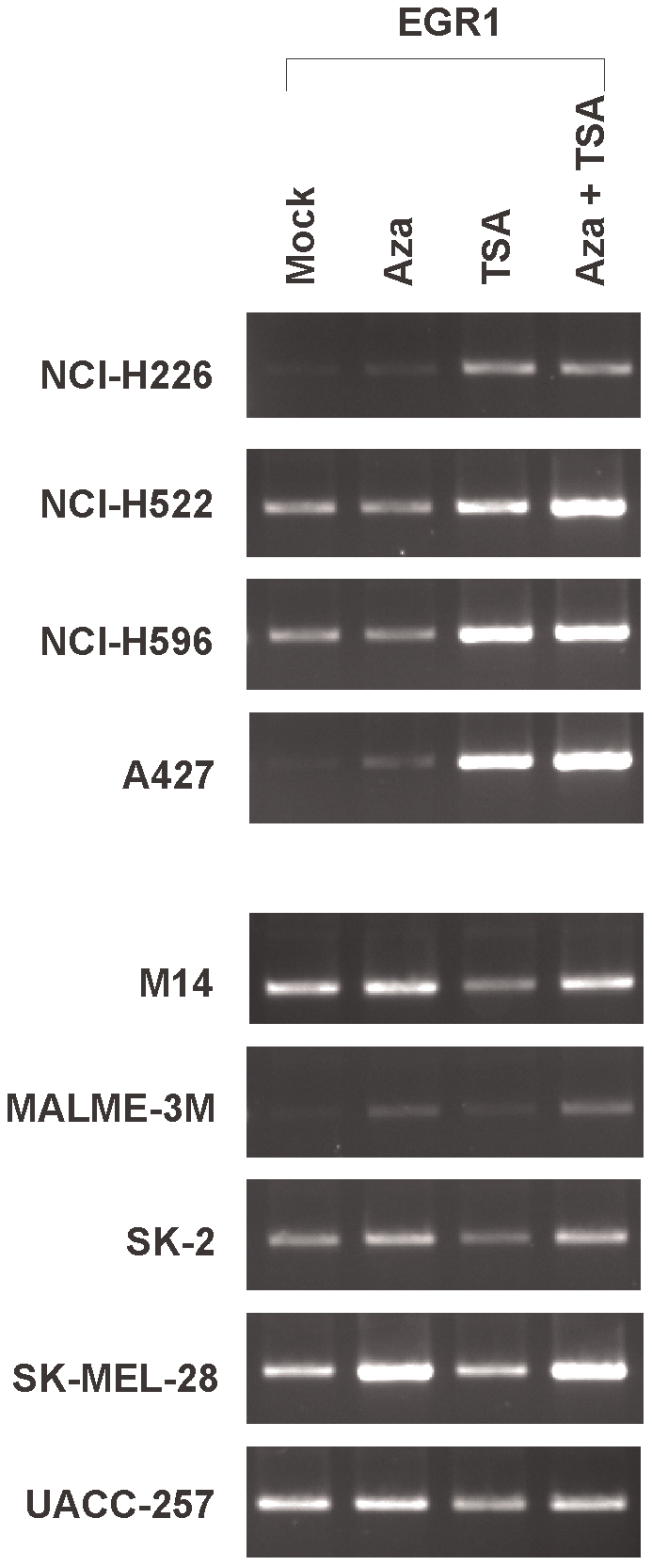
*EGR1* displays an expression pattern similar to that of *MIG-6* in lung cancer and melanoma cell lines upon 5-aza-dC and TSA treatment. The expression of *EGR1* was determined by RT-PCR. (A) TSA induced *EGR1* expression in the NCI-H226, NCI-H522, NCI-H596, and A427 lung cancer cell lines. (B) 5-Aza-dC induced *EGR1* expression in the M14, MALME-3M, SK-2, SK-MEL-28, and UACC-257 melanoma cell lines. *GAPDH* expression was used as an internal control (see Figure 3).

Among the up-regulated genes were those coding for transcription factors such as EGR1 and STAT1, the MIG-6-inducible gene *HBEGF*, and genes coding for histone proteins (Figure 9A). Even though those genes were differentially expressed in A427 and M14 cells (Figure 9A), further analyses revealed that *EGR1* (but not several other genes we examined) displayed an expression pattern similar to that of *MIG-6* across the four lung cancer cell lines and five melanoma lines (Figure 10). Thus, *MIG-6* was not the only gene differentially regulated in the lung cancer and melanoma cells. Perhaps there are tissue-specific factors (either transcription factors or transcription factor co-activators/co-repressors) that respond differently to 5-aza-dC and TSA, leading to differential induction of *MIG-6* and *EGR1* in lung cancer and melanoma cells.

## Discussion

*MIG-6*, a tumor suppressor gene, has been found down-regulated in many human cancers. To determine if down-regulation of *MIG-6* expression was affected by epigenetic modification in its promoter, we treated lung cancer and melanoma cell lines with inhibitors of methylation and histone deacetylation and then determined how those inhibitors influenced *MIG-6* expression. Intriguingly, we found that DNMT inhibitor 5-aza-dC specifically induced *MIG-6* expression in melanoma cells but not in lung cancer cells, while the HDAC inhibitor TSA induced the reverse pattern (Figure 2 and 3). Despite both inductions being regulated at transcriptional level, we were surprised to find that the *MIG-6* promoter was neither hypermethylated nor directly affected by histone deacetylation (Figure 4 and 5), indicating that an indirect mechanism might be responsible for differential induction. In fact, 5-aza-dC has also been reported to induce the expression of several other genes whose promoters are not directly affected by methylation in leukemia cells [26], suggesting that 5-aza-dC might have a broader influence on regulating gene expression via a methylation-independent manner.

Many DNMT inhibitors and HDAC inhibitors are currently in clinical trials for their anti-cancer properties [27], [28], [29]. Even though most of these epigenetic drugs are still in early development and the prospects for them to be used clinically for cancer treatment remain to be evaluated, that evaluation will depend on our understanding of how they work and what outcomes might be expected. 5-Aza-dC and TSA are viewed as potent and specific inhibitors for methylation and histone deacetylation, respectively [27], [28], [30], and they have been widely used for investigating epigenetic alteration of many tumor suppressor genes. These inhibitors usually cause global changes in gene expression by remodeling chromatin via directly converting methylated DNA to unmethylated DNA or unacetylated histones to the acetylated state, thereby allowing easy access of the transcription machinery to gene promoters. However, some inhibitors might be doing more, and their anti-cancer properties could be much more complicated. For instance, many non-histone cellular proteins such as transcription factors are also substrates of HDAC, and their transcriptional activities could be affected by the HDAC inhibitor TSA as well [29].

Most tumor suppressor genes are epigenetically silenced by either DNA methylation and/or histone deacetylation in their promoters [25]. To our knowledge, there is no report showing that the expression of such genes can be differentially regulated by inhibitors of methylation or histone deacetylation in a cancer-specific fashion without having epigenetic modifications in the promoter. The regulation of *MIG-6* by these inhibitors, as we show here, unveils a novel mechanism by which a tumor suppressor gene can be epigenetically silenced in an indirect and tissue-specific manner. Our luciferase reporter assay results indicated that the regulation of *MIG-6* expression in melanoma and in lung cancer was most likely mediated by different factors. We have identified a minimal TSA response element in exon 1 of *MIG-6* proximal to its promoter (Figure 7 and 8), while location of the 5-aza-dC response element is still uncertain (Figure 6).

**Figure 11:**
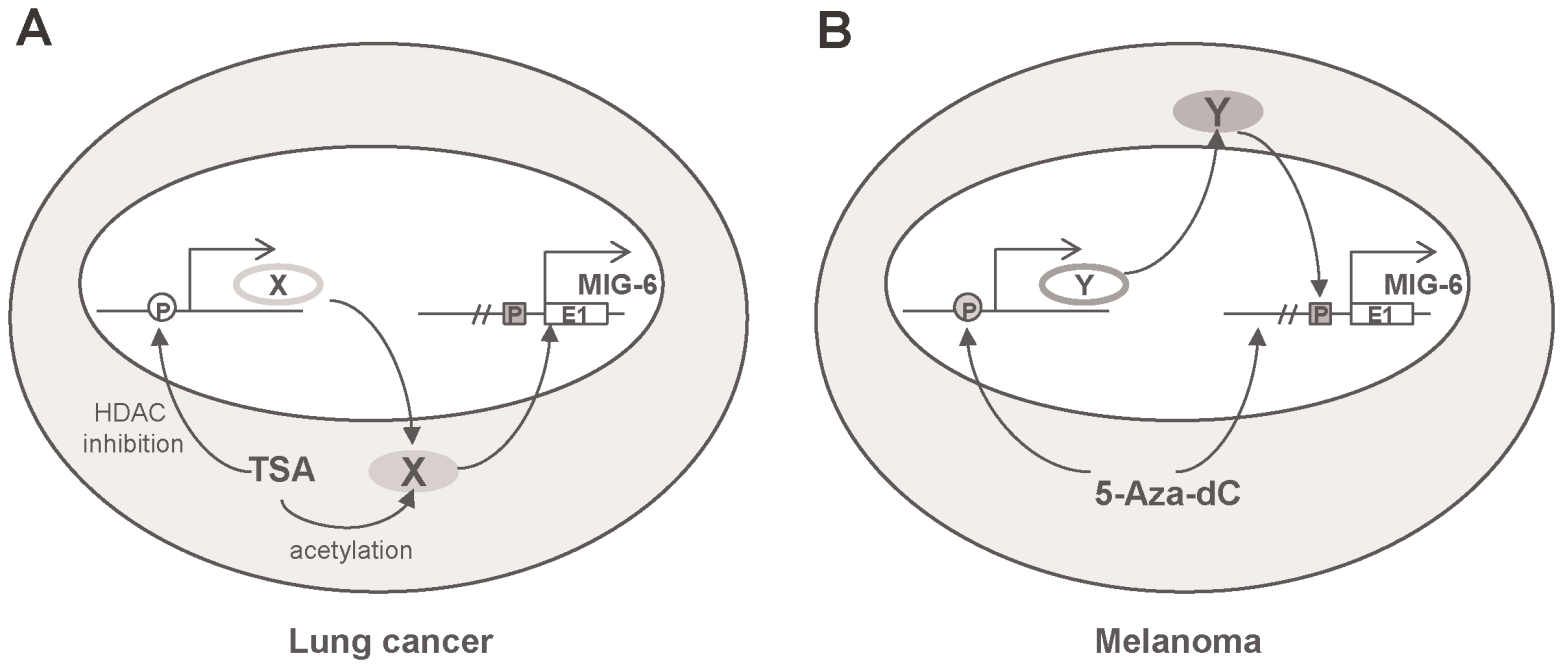
Working models for epigenetic regulation of *MIG-6* expression by 5-aza-dC and TSA. (A) In lung cancer cells, TSA might indirectly regulate *MIG-6* through two mechanisms. The first could be that TSA inhibits histone deacetylation in the promoter of gene *X*, resulting increased production of X protein which enhances *MIG-6* gene expression by associating with the TSA-response element in exon 1. The second might be that direct acetylation of protein X influenced by TSA results in increased transcription of *MIG-6*. (B) In melanoma cells, the regulation of *MIG-6* expression by 5-aza-dC might be direct or indirect. 5-Aza-dC might inhibit DNA methylation in the promoter of gene *Y*, leading to up-regulation of its product and thus indirectly enhancing *MIG-6* expression; or it might directly inhibit DNA methylation outside the tested 1.383-kb *MIG-6* promoter regulatory region, allowing easy access of a transcriptional co-activator to enhance *MIG-6* expression. “P” indicates the promoter of each gene; “E1” indicates exon 1 of the *MIG-6* gene.

We speculate that the TSA response element in the *MIG-6* gene is most likely regulated by a factor whose expression is affected by histone deacetylation in its promoter or whose protein activity is directly regulated by acetylation/deacetylation (Figure 11A). This factor would be activated in lung cancer cells upon TSA treatment, but not in melanoma cells. Within the minimal TSA-response element that we identified in *MIG-6* gene exon 1 (Figure 7), there are putative DNA binding sequences for the transcription factor activator protein-2 (TFAP2), which has five family members and binds to the consensus sequence 5′-GCCNNNGGC-3′ [31], [32]. When the putative TFAP2 binding sites were mutated, we observed a significant drop in TSA-responsiveness (Figure 8), indicating that those sequences are crucial for TSA-mediated regulation. It will be interesting to see if TFAP2 or other factor(s) binds to those sequences and regulates MIG-6 gene expression. As for 5-aza-dC, its response element is likely outside the tested 1.383-kb *MIG-6* promoter regulatory region (Figure 6); that is, it is either directly affected by methylation in its DNA sequences or is indirectly mediated by another transcriptional regulator whose promoter is modified by methylation in melanoma cells (Figure 11B). Extensive studies will be required to determine what those factors are and how they control *MIG-6* expression.

Cancer-type regulation of gene expression by inhibitors of methylation and histone deacetylation is not unique to *MIG-6*. Other genes such as *EGR1* [33] are also differentially regulated in lung cancer and melanoma cells by those inhibitors (see Figures 9 and 10). It remains to be determined whether–like the *MIG-6* promoter–the *EGR1* promoter is neither hypermethylated nor affected by histone deacetylation in those cells. If these characteristics are the same in the two promoters, it will be interesting to see if they are regulated by same factor(s) or via different mechanisms.

We report here that *MIG-6* expression is differentially regulated by inhibitors of methylation and histone deacetylation in lung cancer and melanoma cells without physical epigenetic alterations in its promoter. *MIG-6* (and possibly *EGR1*) may serve as valuable biomarkers for determining the sensitivity/suitability of a cancer type for treatment with DNMT and/or HDAC inhibitors in the clinic.

## Materials and Methods

### Human Cell Lines

The human lung cancer cell lines A427, NCI-H292, NCI-H2122, NCI-H596, and SK-MES-1 were obtained from American Type Culture Collection (Manassas, VA). EBC-1 was from the Health Science Research Resources Bank (Tokyo, Japan). NCI-H226 and NCI-H522 were obtained from NCI-60 cell lines (NCI-Frederick). They were cultured in RPMI 1640 supplemented with 10% fetal bovine serum (FBS) and 1% penicillin/streptomycin. The human melanoma cell lines A375, C8161R, MALME-3M, M14, SK-2, SK-MEL-28, SK-MEL-103, SK-MEL-147, UACC-62, and UACC-257 were kindly provided by Dr. Matthew VanBrocklin (Nevada Cancer Institute, Las Vegas, NV) [34] and maintained in RPMI supplemented with 5% FBS and 1% penicillin/streptomycin.

### Plasmids

A series of DNA fragments derived from the *MIG-6* promoter regulatory region were inserted into *Bgl*II and *Kpn*I sites in the promoter-less luciferase reporter pGL3-Basic (Promega, Madison, WI) to create the plasmids pGL3-P(−1076/+307),–P(−533/+307),–P(−106/+307),–P(−76/+307),–P(−76/+255),–P(−76/+245),–P(−76/+177),–P(−76/+139),–P(−76/+50),–P(−76/+31),–P(−76/+20),–P(−76/+10), and–P(−76/−1). All inserted fragments were amplified by polymerase chain reaction (PCR) using *Pfu* turbo DNA polymerase (Stratagene, La Jolla, CA), and the resulting plasmids were sequenced to confirm the accuracy of the inserts.

All pGL3-P(−76/+50) mutant reporters were created using a QuikChange Site-Directed Mutagenesis Kit (Stratagene) by mutating each of six original nucleotides into an *EcoR*I restriction enzyme site (GAATTC), which was confirmed by sequencing. The primers used for creating each mutant reporter are as follows: for pGL3-P(−76/+50)m3, 5′-TAGCGGGAGCGCGAGAATTCAAGAGGCGCCTGCG-3′ (sense) and 5′-CGCAGGCGCCTCTTGAATTCTCGCGCTCCCGCTA-3′ (antisense); for–P(−76/+50)m4, 5′-GAGCGCGAGCCAGCAGAATTCGCCTGCGCAGATCT-3′ (sense) and 5′-AGATCTGCGCAGGCGAATTCTGCTGGCTCGCGCTC-3′ (antisense); for–P(−76/+50)m5, 5′-AGCCAGCAAGAGGCGAATTCGCAGATCTGCGATCT-3′ (sense) and 5′-AGATCGCAGATCTGCGAATTCGCCTCTTGCTGGCT-3′ (antisense); for–P(−76/+50)m6, 5′-GCGGCGACGGCGGTCGAATTCGAGGCAGAGTGCTA-3′ (sense) and 5′-TAGCACTCTGCCTCGAATTCGACCGCCGTCGCCGC-3′ (antisense); for–P(−76/+50)m7, 5′-CGGCGGTCCGGTGCGAATTCGAGTGCTAGCGGGAG-3′ (sense) and 5′-CTCCCGCTAGCACTCGAATTCGCACCGGACCGCCG-3′ (antisense); for–P(−76/+50)m8, 5′-CCGGTGCGAGGCAGAATTCTAGCGGGAGCGCGA-3′ (sense) and 5′-TCGCGCTCCCGCTAGAATTCTGCCTCGCACCGG-3′ (antisense); for–P(−76/+50)m9, 5′-GAGGCAGAGTGCTAGAATTCGCGCGAGCCAGCAAG-3′ (sense) and 5′-CTTGCTGGCTCGCGCGAATTCTAGCACTCTGCCTC-3′ (antisense); for–P(−76/+50)m10, 5′-GAGTGCTAGCGGGAGAATTCGCCAGCAAGAGGCGC-3′ (sense) and 5′-GCGCCTCTTGCTGGCGAATTCTCCCGCTAGCACTC-3′ (antisense); and for–P(−76/+50)m11, 5′-GCGAGCCAGCAAGAGAATTCTGCGCAGATCTGCG-3′ (sense) and 5′-CGCAGATCTGCGCAGAATTCTCTTGCTGGCTCGC-3′ (antisense). The underline indicates the *EcoR*I site for each mutant.

### Western Blot Analysis

To prepare protein lysates, cells were treated with or without 5-aza-dC (10 µM) for 3 d or TSA (1 µM) for 1 d in complete growth medium. For the combination of the two, cells were treated with 5-aza-dC for 2 d, followed by TSA treatment for 1 d. Whole cell lysates were extracted in RIPA buffer (20 mM Tris-HCl pH 7.5, 150 mM NaCl, 1% NP 40, 0.5% deoxycholate, 0.1% SDS, 1 mM EDTA, 50 mM NaF, 1 mM sodium orthovanadate, and Complete Proteinase Inhibitor Cocktail tablets), quantified using a DC Protein Assay Kit (Bio-Rad, Hercules, CA), and adjusted with equal volume of 2× Laemmli Sample Buffer (Sigma, Saint Louis, MO). The protein was run in a 10% Tris-glycine gel (Invitrogen, Grand Island, NY), transferred onto a PVDF membrane, and probed with anti-MIG-6 [11] or anti-β-actin antibody (Sigma).

### RNA Preparation and RT-PCR Analysis

Using TRIzol reagent (Invitrogen), total RNA was isolated from each cell line treated with 5-aza-dC and/or TSA and from controls, as detailed above. For reverse transcription (RT)-PCR, first-strand cDNA was first synthesized from 1 µg RNA using SuperScript II reverse transcriptase (Invitrogen), followed by PCR amplification using 5% cDNA for each reaction. Following are the primers used for amplification of each gene: for *MIG-6*, 5′-ATGTCAATAGCAGGAGTTGCTG-3′ (sense) and 5′-GTCTAAGGAGAAACCACATAGG-3′ (antisense); for *GAPDH*, 5′-AACGGATTTGGTCGTATTGGGC-3′ (sense) and 5′-GCTTCACCACCTTCTTGATGTC-3′ (antisense); and for *EGR1*, 5′-AACTGTGTCCCCTGCAGCTCCA-3′ (sense) and 5′-CCACAAGGTGTTGCCACTGTTG-3′ (antisense).

### Bisulfite DNA Sequencing

Genomic DNAs isolated from each cell line were subjected to sodium bisulfite conversion of unmethylated cytosine in DNA using EpiTect Bisulfite Kit (QIAGEN, Valencia, CA) according to the manufacturer's protocol. Bisulfite-treated DNAs were then used for nested PCR using following primers: 5′-GGAGAGGAAAAAATATATAATTTTGTT-3′ (forward primer p1) and 5′-TCTCCCTCCATCCCAAAAACTC-3′ (reverse primer p5) for the first round of PCR, and for the second round, forward primer p1 and 5′-TAACCCTCCCCACCCCCTCAAC-3′ (reverse primer p4). The second round PCR products (596 bp) were then cloned into the TOPO TA Cloning vector (Invitrogen). For each cell line, 10 different colonies were picked and the inserts were sequenced. Direct sequencing of the second round PCR products was also performed. The MethPrimer program (www.urogene.org/methprimer) was used for analyzing CpG islands in the *MIG-6* gene.

### Chromatin Immunoprecipitation (ChIP) Assay

The ChIP assay was performed using a Chromatin Immunoprecipitation Assay Kit (Upstate, Lake Placid, NY) according to the manufacturer's instructions. Briefly, cells were treated with or without TSA (1 µM) for overnight, and then histones in the cells were cross-linked to DNA by 1% formaldehyde treatment for 10 min. After crosslinking, cells were collected, resuspended in SDS lysis buffer, and sonicated to shear the DNA. The lysates were centrifuged to clear debris, and the supernatants were collected and diluted with 10-fold ChIP Dilution Buffer containing protease inhibitor cocktail (Roche, Indianapolis, IN). The diluted supernatants were then incubated with either anti-acetyl histone H3 antibody (Upstate) or normal rabbit serum (as negative control) overnight at 4°C, followed by a 1 h incubation with Protein A agarose/salmon sperm DNA for immunoprecipitation. After extensive washing, the precipitated antibody/histone/DNA complex was eluted in freshly prepared elution buffer (1% SDS, 0.1 M NaHCO_3_), and the histone-DNA crosslinks were reversed by heating at 65°C for 4 h. Precipitated DNAs were recovered by phenol/chloroform extraction and ethanol precipitation and then stored at −20°C until used for PCR. Primers used for PCR were as follows: for the *MIG-6* gene promoter, 5′-AGACGCCTCTCCGGGAGAC-3′ (forward) and 5′-ATAGGCCGCCGGGCCGTGA-3′ (reverse); and for the *GAPDH* gene promoter, 5′-TCGGTGCGTGCCCAGTTGAACC-3′ (forward) and 5′-ATGCGGCTGACTGTCGAACAGG-3′ (reverse).

### Microarray Analysis

Total RNA was isolated from A427 and M14 cells treated with 5-aza-dC and/or TSA and from controls, as described above. Agilent 44K One-Color Microarrays (Agilent, Santa Clara, CA) were used for detection and analyses of gene expression.

### Luciferase Reporter Assay

The day before transfection, 5×10^4^ cells were seeded in each well of a 96-well plate. The firefly luciferase reporter plasmid pGL3-luc or its derivative (100 ng) was co-transfected with pU6B-*Renilla* reporter (5 ng) using Lipofectamine 2000 (Invitrogen) overnight. The cells were then treated with 5-aza-dC (10 µM) for 2 d or TSA (1 µM) for 1 d. The luciferase reporter activities were assayed using the Dual-Luciferase Reporter Assay System (Promega) and measured using an EnVision 2104 Multilabel Reader (PerkinElmer, Waltham, MA). The firefly luciferase activities were normalized for analyses using *Renilla* luciferase activities.
